# Oral Contraceptive Use and Breast Cancer Risk Assessment: A Systematic Review and Meta-Analysis of Case-Control Studies, 2009–2020

**DOI:** 10.3390/cancers13225654

**Published:** 2021-11-12

**Authors:** Agnieszka Barańska, Agata Błaszczuk, Wiesław Kanadys, Maria Malm, Katarzyna Drop, Małgorzata Polz-Dacewicz

**Affiliations:** 1 Department of Medical Informatics and Statistics with E-Learning Lab, Medical University of Lublin, 20-090 Lublin, Poland; maria.malm@umlub.pl; 2 Department of Virology with SARS Laboratory, Medical University of Lublin, 20-059 Lublin, Poland; agata.blaszczuk@umlub.pl (A.B.); malgorzata.polz.dacewicz@umlub.pl (M.P.-D.); 3 Specialistic Medical Center Czechow, Gynecology Unit, 20-848 Lublin, Poland; wieslaw.kanadys@wp.pl; 4 Department of Language, Rhetoric and Media Law, John Paul II Catholic University, 20-950 Lublin, Poland; drop.katarzyna@kul.pl

**Keywords:** oral contraceptives, breast cancer, risk, risk factors, breast malignancies

## Abstract

**Simple Summary:**

Breast cancer (BrCa) is a heterogeneous disease and has important variability according to ethnicity and race with respect to incidence, clinical characteristics, and prognosis. Numerous epidemiological studies indicate that BrCa and it’s also related to environmental factors. We, therefore, undertook a systematic review of the literature regarding BrCa risk in women who used OCs based on case-control studies carried out in the years 2009–March 2020 and then performed a meta-analysis of relevant data. Increased BrCa risk was associated with early menarche, nulliparous, non-breastfeeding, older age at first parity, postmenopause, obesity, smoking, and family history of BrCa.

**Abstract:**

To perform a meta-analysis of case-control studies that addressed the association between oral contraceptive pills (OC) use and breast cancer (BrCa), PubMED (MEDLINE), Embase, and the Cochrane Library were searched to identify case-control studies of OC and BrCa published between 2009 and 2020. We used the DerSimonian–Laird method to compute pooled odds ratios (ORs) and confidence intervals (CIs), and the Mantel–Haenszel test to assess the association between OC use and cancer. Forty-two studies were identified that met the inclusion criteria and we included a total of 110,580 women (30,778 into the BrCa group and 79,802 into the control group, of which 15,722 and 38,334 were using OC, respectively). The conducted meta-analysis showed that the use of OC was associated with a significantly increased risk of BrCa in general, OR = 1.15, 95% CI: 1.01 to 1.31, *p* = 0.0358. Regarding other risk factors for BrCa, we found that increased risk was associated significantly with early menarche, nulliparous, non-breastfeeding, older age at first parity, postmenopause, obesity, smoking, and family history of BrCa. Despite our conclusion that birth control pills increase the cancer risk being supported by extensive previous studies and meta-analyzes, further confirmation is required.

## 1. Introduction

Among malignant tumors, BrCa is the leading type of cancer that affects women in most countries. According to the predictions of the WHO’s International Agency for Research on Cancer, the specific age-standardized incidence rate for cancer of the female breast worldwide is 46 cases per 100,000 women. Herein, incidence rates are elevated in Australia/New Zealand (94), Western Europe (93), Northern Europe (90), and North America (85). In contrast, incidence rates in sub-Saharan African regions, particularly in Eastern (30) and Middle Africa (28), as well as South-Central Asia (26), are considerably lower [1].

BrCa is a heterogeneous disease and has important variability according to ethnicity and race with respect to incidence, clinical characteristics, and prognosis. The majority of BrCa cases are sporadic; however, it is estimated that approximately 5–10% have a genetic predisposition related to, among others, family cancer histories for first-degree relatives or genetic mutation carrier status [2,3]. Of these, 40% are due to mutations in either of the two tumor suppressor genes BRCA (BReast CAncer gene) 1 and 2, which are localized on chromosomes 17g21 and 13q12.3, respectively [4,5].

Furthermore, variations in the risk of developing BrCa have been reported in relation to molecular subtypes, defined by estrogen receptor (ER), progesterone receptor (PR), and human epidermal growth factor receptor 2 (HER2) expression [6,7,8].

The variations in the incidence of BrCa between countries may depend on differences in the incidence and distribution of risk factors, as well as the level of early detection and screening carried out in these countries. Numerous epidemiological studies indicate that BrCa is related to reproductive factors, such as early menarche below 12 years of age, late menopause above 55 years of age, nulliparity, miscarriages before the first full-term pregnancy, late age at first birth, infertility, and hormone usage. BrCa is also related to environmental factors that include high socio-cultural level, obesity, selected dietary habits, alcohol consumption, low physical activity, and exposure to ionizing radiation (used for therapeutic purposes) [9,10,11,12,13,14,15].

Our first meta-analysis on the effects of OC on BrCa risk covered the period 1960–2010 [16]. The meta-analysis included studies that examined the mixture of pills from four generations: the first-generation progestin included norethindrone, lynestrenol, ethynodiol diacetate, and norethisterone; the second-generation included levonorgestrel, and norgestrel, introduced in the 1990s; the third-generation included desogestrel, gestodene, and norgestodene introduced in the 1990s; and the last approved fourth-generation ethinyloestradiol/drospirone and estradiol valerate/dienogest. The continuation of the previous publication is a meta-analysis covering the period from 2009 to 2020. Third- and fourth-generation OC dominated this meta-analysis. Both publications constitute a research cycle for assessing the impact of contraception on BrCa. Although the relationship between OC use and BrCa risk has been extensively studied, this topic remains an important research area. Indeed, this issue is still pending final explanation [17,18]. We, therefore, undertook a systematic review of the literature regarding BrCa risk in women who used OCs based on case-control studies carried out in the years 2009–March 2020 and then performed a meta-analysis of relevant data.

## 2. Materials and Methods

### 2.1. Search Strategy and Selection Criteria

A systematic review and meta-analysis of published case-control studies assessing the impact of OC on the risk of female BrCa development was performed based on the guidance “Preferred Reporting Items for Systematic Reviews and Meta-analysis for 2015 protocols (PRISMA-P 2015)” [19]. Our research was limited to articles published from 2009 to March 2020.

The contents of the medical databases of PubMed (MEDLINE), Embase, and the Cochrane Library were reviewed to identify studies related to the assumptions of our work. To provide a complete overview of the available relevant studies, we additionally scrutinized references to previously published review articles, meta-analyses, and other publications.

The following inclusion criteria were established in the selection of studies: (i) full-text articles published between 2009 and 2020; (ii) case-control design (design (population- and hospital-based)); (iii) data on the correlation between OC use and BrCa; (iv) articles written in English; (v) data included in the articles were sufficient to calculate the odds ratio (OR) and 95% confidence interval (CI); (vi) at least 20 subjects use of OC included in the case group; and (vii) if there was an overlap in the cases included, only the latest and most comprehensive data were selected. The exclusion criteria were as follows: insufficient quantitative data; the results were reported as graphics; duplicate reports; and articles published in languages other than English [20].

Two reviewers independently checked the titles and abstracts of all papers retrieved from databases. They next extracted relevant study data from the full-text papers selected for inclusion. Any disagreements were resolved by consensus. Articles were initially evaluated according to title and/or abstract. Subsequently, the decision was made to include or exclude after independent and double analysis and full tests of selected research. After approval, the studies were qualified for meta-analysis and collection of data on their clinical and methodological characteristics.

### 2.2. Data Extraction

The following data were extracted for each study: (i) clinical and methodological study characteristics such as last name of the first author, publication year, the country in which the study was performed, name of the study, years of data collection, number of cases and control subjects, and source of cases; (ii) information on the usage of OC in both groups (ever/never); and (iii) BrCa incidence depending on menarche, parity, breastfeeding, menopausal status, family history of BrCa, nutritional status, diabetes, and tobacco smoking.

### 2.3. Assessment of Study Quality

Methodological quality was evaluated by means of the Newcastle–Ottawa scale (NOS) for quality assessment of the included studies. In this evaluation, scores from 0 to 3, from 4 to 6, and from 7 to 9 were given for low, medium, and high quality, respectively [21]. With this tool, each study in the meta-analysis was assessed in three separate categories: selection of cases and controls, comparability of cases and controls on the basis of the design or analysis, and ascertainment of exposure. The NOS quality stars ranged between 4–8, and the average score was 5.76 for included studies. Fifteen (35.71%) studies were regarded as high quality (NOS ≥ 7 points), and 27 (64.29%) studies were regarded as medium quality (NOS ≥ 4 points).

### 2.4. Statistical Analysis

The meta-analysis of summary statistics from individual studies was performed through STATISTICA 13.3 software (StatSoft Poland, Krakow, Poland), using the Medical Package program. For each study, we constructed separate two-by-two (2 × 2) contingency tables to calculate the odds ratios (OR) and 95% confidence intervals (CIs), cross-classifying OC users and occurrence of BrCa. The Mantel-Haenszel test was used to assess the relationship between OC use and BrCa. The meta-analysis combining the ORs across studies was conducted using the DerSimonian–Laird random-effects model [22]. The random-effects meta-analysis model was used due to the diversity of research in terms of, for example, design and population. In the random-effects model, it is assumed that there is no common effect size for independent studies. Instead, each study is assumed to have a different population effect size, which is a random variable and has a normal distribution. Therefore, there is a difference between the size of the effects of individual studies. Thus, the variance of the effect size in the random-effects model is the sum of the variance within and between studies. The weighting of the studies in the meta-analysis was calculated on the basis of the inverse of the sum of ‘within study’ and between studies variances.

Heterogeneity was assessed graphically by employing a forest plot and statistically by applying the Q test and I^2^ index. I^2^ values of 25%, 50%, and 75% have been regarded as respectively representing low, moderate, and high heterogeneity between studies [23].

Publication bias was explored via funnel plots and estimated using Begg’s and Egger’s tests [24,25]. For all the analyses, a forest plot was generated to display results, whereby diamonds represent study-specific odds ratios; 95% CIs for individual studies are represented by horizontal lines.

### 2.5. Subgroup Analysis

Prespecified subgroup analyses were carried out to identify the sources of heterogeneity between studies in accordance with reproductive factors: age of menarche (≤12 years/>12 years), parity (nulliparous/parous), age at first pregnancy (≥25 years/>25 years; ≥30 years/<30 years), breastfeeding (no/yes) and menopausal status (pre-/post-menopausal); as well as personal risk factors: the period of OC use (≤5 years; >5 years), body mass index (≥30 kg/m^2^/<30 kg/m^2^), diabetes (yes/no), tobacco smoking (ever/never), and family history of BrCa (yes/no).

## 3. Results

**Figure 1:**
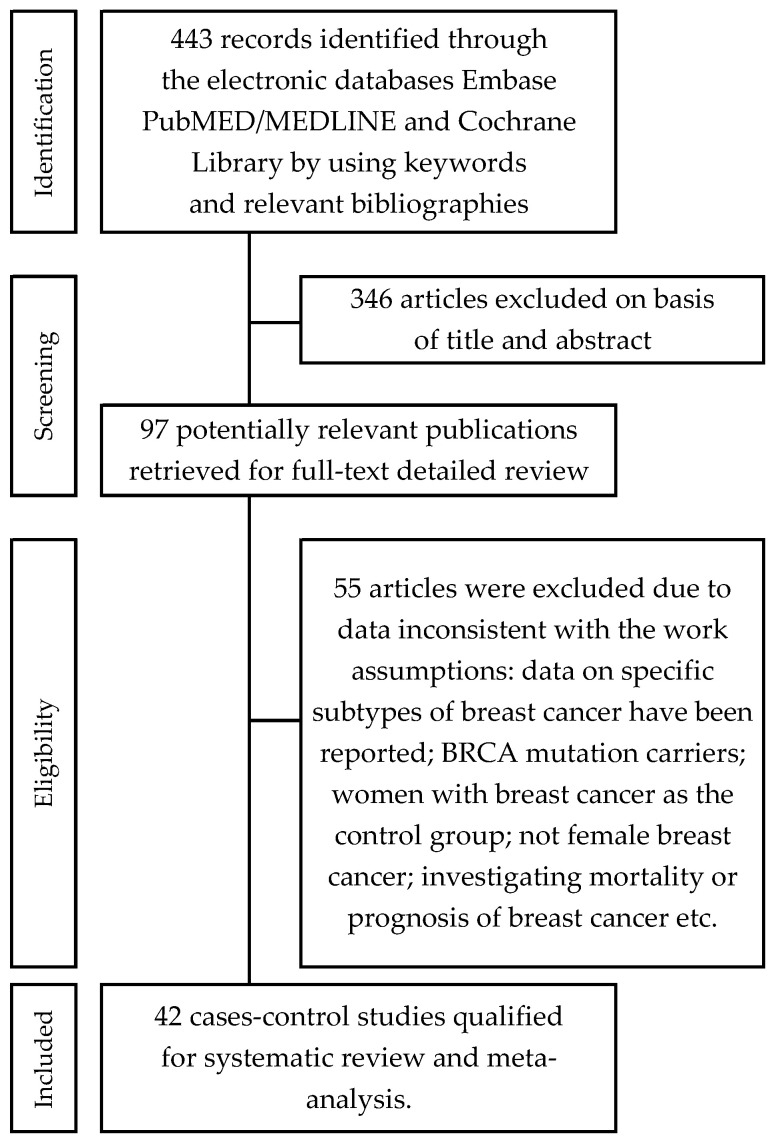
Flowchart of the selection procedure for studies included in the current review and meta-analysis.

We identified 443 references through the medical electronic databases PubMed (MEDLINE), Embase, and the Cochrane Library up to March 2020. We excluded 346 citations on the basis of titles and abstracts, and 99 citations after reviewing the full texts. These studies were excluded because the full text was not available in the English language, they were letters to the editor, commentaries, review articles, of inappropriate designs (studies of cross-sectional, randomized controlled trials, prospective/retrospective cohort studies), or BrCa was only reported. Moreover, exclusion occurred because the subjects were BRCA mutation carriers, women with BrCa were the control group, the papers were not about female BrCa, the researchers were investigating mortality or prognosis of BrCa, etc. Eventually, 42 case-control studies were qualified in the review and meta-analysis (Figure 1).

In total, 110,580 women (30,778 in the group with BrCa and 79,802 in the control group, of which 15,722 and 38,334 used of OC, respectively) were included in the meta-analysis. Of the studies included:

- seven (7) case-control studies were conducted in the Americas,
- six (6) in Africa,
- sixteen (16) in the Middle East,
- eleven (11) in Asia,
- one (1) in Europe,
- one (1) was an international, multicenter research study.

Characteristics of selected works are shown in Table 1.

**Figure 2:**
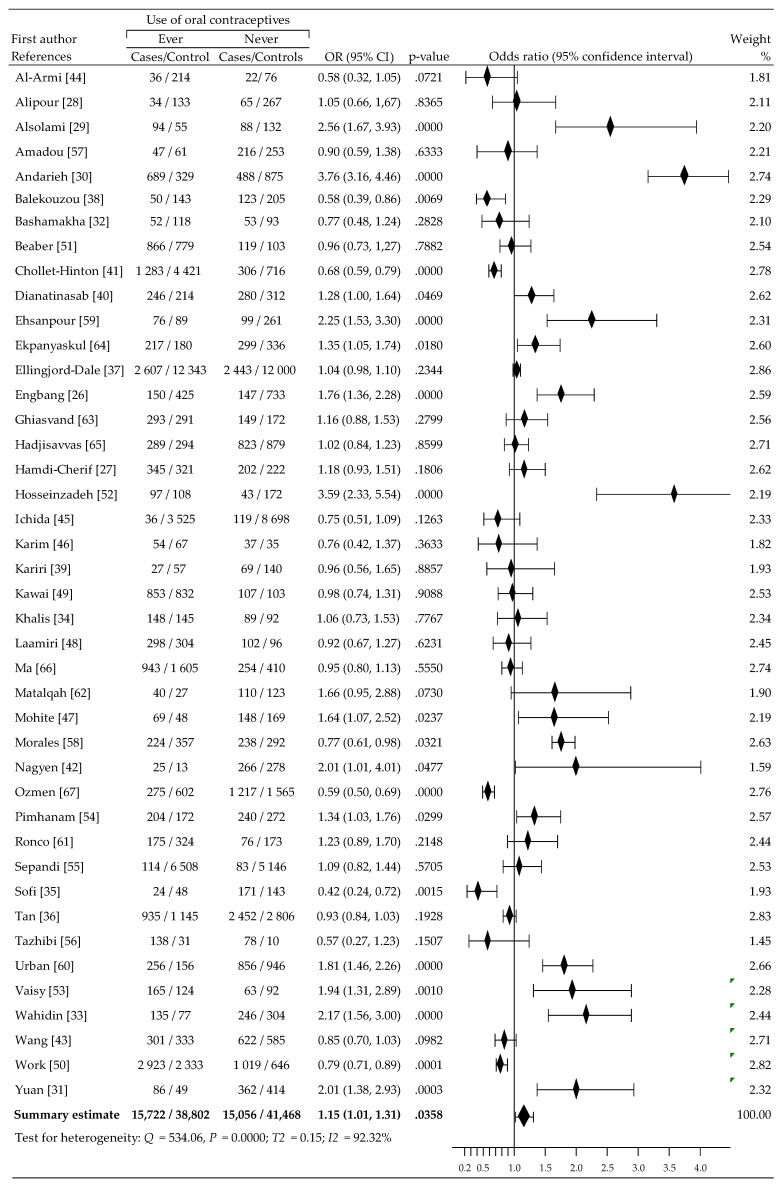
A forest plot and the summary odds ratios for the relationship between BrCa risk and ever OC use: case-control studies conducted 2009–2020 in alphabetical order.

Figure 2 shows the change incidence of BrCa risk related to OC use in each study and overall. An increase in the risk of BrCa among women using OC was reported in 23 of 42 trials, including the increase being statistically significant at 14 trials. In comparison, a decrease in the risk of BrCa was observed in participants not using OC in 19 of 42 studies, with the reduction being statistically significant in 6 trials. Meta-analysis using a random-effects model show moderate, statistically significant increase of BrCa risk: OR = 1.15 (95% CI, 1.01 to 1.31), *p* = 0.0358. This was accompanied by high heterogeneity: I^2^ = 92.32%.

**Figure 3:**
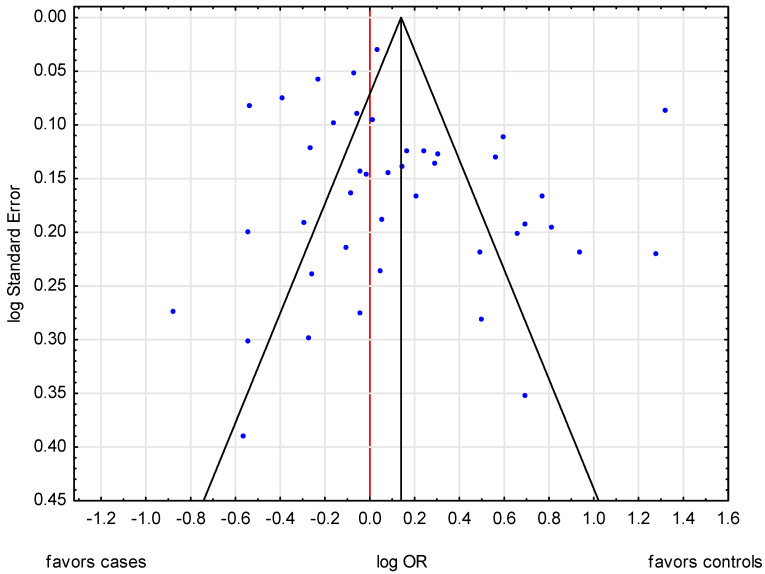
A funnel plot of publication bias in the meta-analysis of the relationship between BrCa risk and ever use of OC.

No obvious evidence of publication bias was detected by inspection of the funnel plot (Figure 3). Moreover, the Begg and Mazumdar’s test for rank correlation did not indicate evidence of publication bias. Accordingly, Kendall’s tau = 0.0580; Z = 0.5202; *p* = 0.6029; similarly, Begg and Egger’s test: b0 = 1.2587 (95% CI: −0.6964 to 3.2138); t = 1.3012, *p* = 0.2006.

A summary of the results of the studies providing data for the assessment of BrCa risk is shown in Table 2. For the period of contraceptive usage, as indicated through meta-analysis, 13 studies [28,33,37,38,41,48,49,50,51,53,59,62,66] showed a slight statistical insignificant increase of BrCa risk in women taking OC longer than 5 years (OR = 1.05, 95% CI: 0.88 to 1.25, *p* = 0.5787) as well as a slight statistically insignificant decrease in the risk of BrCa in women taking OC for less than or equal to 5 years (OR = 0.92, 95% CI: 0.77 to 1.10, *p* = 0.3674).

Reproductive factors included age at menarche, parity, age at first birth, breastfeeding and menopausal status. The earlier age of menarche (≤12 years) was associated with a statistically significant higher BrCa risk (OR = 1.18, 95% CI: 1.07 to 1.31, *p* = 0.0016), as shown in the meta-analysis of 29 studies [26,27,30,32,33,34,35,36,37,38,39,41,43,44,47,48,49,50,51,52,54,55,56,57,58,61,62,64,66]. In addition, analysis of 30 studies of parity [26,27,30,34,35,36,37,38,39,41,42,43,45,47,48,49,50,51,52,54,55,56,57,58,61,62,63,65,66,67] indicated that nulliparous women had a significantly increased risk for BrCa compared to women who gave birth (OR = 1.22, 95% CI: 1.04 to 1.43, *p* = 0.0146). A total of 14 studies [27,35,36,37,39,41,43,49,51,55,62,63,65,67] evaluated the association between first pregnancy above 25 years and risk of developing BrCa. Compared to the first births under the age of 25 years, increased risk was noted (OR = 1.09, 95% CI: 0.94 to 1.25, *p* = 0.2599). What is more, the analysis of six studies [26,48,52,58,64,65] showed a statistically significant higher risk of BrCa in women giving birth for the first time after 30 than before the age of 30 (OR = 3.08, 95% CI: 1.10 to 8.60, *p* = 0.0322). Beyond the aforementioned, in the meta-analysis of 27 studies [26,29,30,32,33,36,37,38,39,41,43,44,45,46,47,48,50,51,52,54,58,61,62,64,65,66,67], non-breastfeeding was associated with a significant increase in the risk for BrCa, in comparison to breastfeeding (OR = 1.36, 95% CI: 1.13 to 1.63, *p* = 0.0010). Finally, the data from 22 studies [26,32,34,35,36,37,39,42,43,44,45,46,47,48,50,52,54,55,58,62,63,64] demonstrated a statistically significant increase in BrCa risk among postmenopausal women (OR = 1.36, 95% CI: 1.14 to 1.63, *p* = 0.0007) when compared to premenopausal women.

Among personal risk factors, the meta-analysis for BMI based on 16 studies [26,27,28,29,32,34,39,41,44,49,51,54,55,57,63,66] indicated a non-significant increase in risk for obese women (OR = 1.19, 95% CI: 0.95 to 1.50, *p* = 0.1289) compared to women who are underweight/normal/overweight. Tobacco use also had significant associations with BrCa risks (OR = 1.52, 95% CI: 1.26 to 1.83, *p* = 0.0000) based on the meta-analysis of 14 studies [26,28,29,30,36,42,49,52,54,58,62,64,66,67]. Likewise, 29 studies [27,28,29,30,32,33,34,35,37,39,41,43,44,45,46,48,49,50,51,52,54,55,57,58,61,63,64,66,67] showed that family history of BrCa in the first or second relatives was significantly associated with higher risk (OR = 1.72, 95% CI: 1.32 to 2.24, *p* = 0.0001). Finally, an analysis of four studies [26,29,32,39] found a slightly increased, but not statistically significant, risk of BrCa in women with diabetes (OR = 1.99, 95% CI: 0.60 to 6.62, *p* = 0.2605).

In most analyses, no obvious evidence of publication bias was detected by inspection of the funnel plot or through statistical tests. There was evidence, however, suggesting publication bias among studies that investigated BrCa, menopausal status (post-/pre-) (Begg–Mazudar’s test: *p* = 0.6769; Egger’s test: *p* = 0.0031), and smoking (yes/no) (Begg–Mazudar’s test: *p* = 0.0381; Egger’s test: *p* = 0.2327).

## 4. Discussion

The presented meta-analysis of the magnitude of effects of OC on BrCa risk revealed a statistically significant slight increase of cancer risk, OR = 1.15 (95% CI; 1.01 to 1.31). The results from our study were, to a large extent, consistent with the results described in previous studies. For example, in 2018, 1245 BrCa cases were found in the analysis of data derived from a prospective national population-based cohort study carried out from 1991 to 2007 of 74,862 Norwegian premenopausal women. These authors saw a statistically insignificant increased risk of BrCa in participants using hormonal contraception. Here, HR (hazard risk) = 1.12, 95%: CI 0.99 to 1.26 [68]. Moreover, over 11,000 BrCa cases were diagnosed from 1995 to 2012 in a large prospective cohort study published in 2017 involving 1.8 million Danish women. The authors of this work noted a moderate significant increase in the relative risk of BrCa (RR = 1.20, 95% CI: 1.14 to 1.26) among users of hormonal contraceptives [69]. Furthermore, the Royal College of General Practitioners Oral Contraception Study conducted in the years 1968–2012 involved a total of 28,993 OC taking women (cancer cases = 4661) and a total of 17,039 women who never took OC (cancer cases = 2341) and found a slight, statistically non-significant increase in the risk of BrCa was evident. Here, IRR (incidence rate ratio) = 1.04, 95% CI: 0.91 to 1.17 [70]. Our previous meta-analysis covering the period 1960–2010 [16] estimated 79 case-control studies conducted between 1960–2010, including a total of 72,030 incidents, histologically confirmed cases of BrCa and 123,650 population/hospital controls. A decrease was observed in cancer risk in OC users before age 25 years (0.91, 0.83–1.00). However, the use of OCs before the first full-term pregnancy significantly increased the risk of BrCa (OR, 1.14, 1.01–1.28, *p* = 0.04), as did OC use longer than 5 years (1.09, 1.01–1.18, *p* = 0.02). Pooled crude odds ratios of BrCa in ever-users of OC was 1.01 [95% confidence interval (CI), 0.95–1.07], compared with never-users. There was no significant increase in risk among premenopausal women (1.06, 0.92–1.22), postmenopausal women (0.99, 0.89–1.10), or nulliparous women (1.02, 0.82–1.26) [16].

Gierisch et al. [71] also conducted a meta-analysis. This work covered fifteen case-control studies (38,682 women) and 8 cohort studies (317,341 women) published between 2000 and 2012. It assessed the association between OC use and BrCa incidence. Overall, in women taking OC, BrCa risk increased slightly, albeit statistically significantly. Here, OR = 1.08 (95% CI, 1.00–1.17). These results were similar to those derived from the meta-analysis by Zhu et al. [72], which included a total of 13 prospective cohort studies on OC use and BrCa risk (11,722 BrCa cases and 859,894 participants) that were identified by searching databases from 1960–2012. In the meta-analysis, the RR (relative risk) of BrCa in women using OC was 1.08 (95% CI: 0.99.17) when compared with never-users. What is more, in a recent publication by Del Puo et al. [73], which analyzed the results of two meta-analyses on the incidence of BrCa among users of hormonal OC, a statistically significant overall increase in the risk of BrCa (OR = 1.08 (95% CI 1.00 to 1.17)) was indicated.

The results of our meta-analyses of potentially modifying risk factors for BrCa indicate that menarche before the age of 12 years, nulliparous, non-breastfeeding, first pregnancy in old age, postmenopause, obesity, smoking, and family history of BrCa significantly increase the risk of development of BrCa. The majority of the above risk factors are associated with a longer duration of estrogen exposure [74,75,76,77,78,79].

We are aware that drawing final conclusions from the results of our meta-analysis requires caution given the number of limitations encountered in its construction. First, we limited the search to studies published in English, and these were identified through electronic databases. The possibility of not reaching out to all publications on this topic may have an effect on the value of the results [80]. Secondly, the studies, including summary estimates, are vulnerable to various types of bias. Retrospective self-reporting of using OC, for example, may be associated with overestimation or underestimation of actuality. Moreover, mistakes may be made in control group recruitment under hospital scenarios. Additionally, mistakes can be made when cases are recruited for study based on the admission of OC use. Thirdly, a lack of a uniform definition of “ever” use of OC exists (this affects the various periods of exposure to OCs), as defined by the times that pill self-administration was begun and ended. Such fuzziness may lead to misclassification, and this, in turn, may weaken the true association between OC use and BrCa affliction (as may occur equally among cases and controls). The limitations should also take into account other coexisting factors, such as the use of different OC and other genetic factors that were not explored in the studies analyzed, such as BRCA1 and BRCA2 mutations (participants were not tested for the presence of mutations in the BRCA1 and BRCA2 genes). Furthermore, a prospective study needs to be conducted to confirm our findings.

## 5. Conclusions

In conclusion, the results from this meta-analysis suggest that OC using is associated with a modest, statistically significant increase in the risk of BrCa. Despite our conclusion that birth control pills increase cancer risk, and that our conclusion is supported by extensive previous studies and meta-analyzes, further confirmation is required.

## Figures and Tables

**Table 1:** Characteristics of the case-control studies included in the meta-analysis on the association between OC use and BrCa risk.

First Autor Pub Year (References)	Region	Recruitment (Year)	Number of Case Subjects (%)	Age (Mean ± SD or Range)	Number of Controls Subjects (%)	Source of Subjects	NOS Score
Engbang 2020 [26]	Duala; Cameroon	2012–2018	297 (50.5)	53.3 ± 12.7	1158 (36.7)	Hospital	8
Hamdi-Cherif 2020 [27]	Setif; Algeria	2012–2017	547 (63.1)	28–77	543 (59.1)	Hospital	4
Alipour 2019 [28]	Golestan; Iran	2004–2008	99 (34.3)	40–75	400 (33.2)	Population	8
Alsolami 2019 [29]	Mahhah; Saudi Arabia	2014–2016	214 (43.9)	57.0 ± 7.3	218 (25.2)	Population	5
Andarieh 2019 [30]	Babolsar; Iran	2014–2016	1177 (58.5)	48.8 ± 8.5	1204 (27.3)	Hospital	7
Yuan 2019 [31]	Chengdu; China	2014–2015	448 (19.2)	43.7 ± 6.1	463 (10.6)	Clinic	5
Bashamakha 2019 [32]	Seiyun; Yemen	2011–2015	105 (49.5)	N/A	210 (55.2)	Population	6
Wahidin 2018 [33]	Jakarta; Indonesia	2018	381 (35.4)	40–49	381 (20.2)	Hospital	4
Khalis 2018 [34]	Fez; Morocco	2014–2015	237 (62.2)	45–54	237 (61.2)	Hospital	6
Sofi 2018 [35]	New Delhi; India	2015–2017	195 (12.3)	45.0 ± 10.0	191 (25.1)	Hospital	7
Tan 2018 [36]	Malaysia	2002–2016	3387 (27.6)	58.0	3951 (29.0)	Hospital	4
Ellingjord-Dale 2017 [37]	Norway	2006–2014	5050 (51.6)	50–69	24,343 (50.7)	Population	8
Balekouzou 2017 [38]	Central African Republic	2003–2015	174 (28.9)	45.8 ± 13.6	348 (41.1)	Population	6
Kariri 2017 [39]	Gaza Strip; Palestine	2014–2015	96 (28.1)	18–60	197 (28.9)	Hospital	7
Dianatinasab 2017 [40]	Shiraz; Iran	2014–2016	526 (46.8)	<40–60>	562 (40.7)	Hospital	7
Chollet-Hinton 2017 [41]	USA	2005	1589 (80.7)	22–75	5137 (86.1)	Community	7
Nguyen 2016 [42]	Hanoi; Wietnam	2007–2013	291 (8.6)	24–65	291 (4.5)	Hospital	7
Wang 2016 [43]	Hong Kong SAR	2011–2015	923 (32.6)	56.0 ± 11.8	918 (36.3)	Hospital	6
Al-Amri 2015 [44]	Riyadh; Saudi Arabia	2013–2014	58 (62.1)	30–69	290 (73.8)	Hospital	6
Ichida 2015 [45]	Tokyo; Japan	2007–2013	155 (23.2)	20–69	12,223 (26.8	Clinic	6
Karim 2015 [46]	Jeddah; Saudi Arabia	2001–2013	92 (58.7)	30–65	100 (67.0)	Clinic	4
Mohite 2015 [47]	Satara district, India	2009–2011	217 (31.8)	40–49	217 (22.1)	Hospital	4
Laamiri 2015 [48]	Rabat; Morocco	2008–2010	400 (74.5)	45.8 ± 11.1	400 (76.0)	Hospital	5
Kawai 2014 [49]	USA	2004–2010	960 (88.9)	20–44	938 (89.1)	Population	6
Work 2014 [50]	USA, Canada, Australia	1995–2004	4011 (72.9)	18–69	2997 (77.8)	Community	6
Beaber 2014 [51]	USA	2004–2010	985 (87.9)	20–44	882 (88.3)	Population	7
Hosseinzadeh 2014 [52]	Tabriz; Iran	2012–2013	140 (69.3)	47.6 ± 10.7	280 (38.6)	Clinic	4
Vaisy 2014 [53]	Urmia; Iran	2013–2014	228 (72.4)	47.63	216 (57.4)	Clinic	5
Pimhanam 2014 [54]	Bangkok; Thailand	2007–2011	444 (45.9)	45.8 ± 10.1	444 (38.7)	Hospital	4
Sepandi 2014 [55]	Shiraz; Iran	2001–2012	197 (57.9)	26–68	11,653 (55.8)	Hospital	4
Tazhibi 2014 [56]	Isfahan; Iran	1999–2010	216 (63.9)	20–75	41 (75.6)	Hospital	5
Amadou 2014 [57]	Mexico	2004–2007	263 (17.9)	35–64	314 (19.4)	Hospital	6
Morales 2013 [58]	Puerto Rico	2005–2009	462 (48.5)	56.4 ± 12.6	649 (55.0)	Hospital	6
Ehsanpour 2013 [59]	Isfahan; Iran	2011	175 (43.4)	<41–60+	350 (25.4)	Clinic	5
Urban 2012 [60]	South Africa	1995–2006	1112 (23.0)	49.0	1102 (14.2)	Hospital	4
Ronco 2012 [61]	Montevideo; Uruguay	2004–2010	251 (69.7)	<30–50≥	497 (65.2)	Hospital	8
Matalqah 2011 [62]	Penang, Malaysia	2009–2010	150 (26.7)	52.8 ± 1.1	150 (18.0)	Population	7
Ghiasvand 2011 [63]	Shiraz; Iran	2005–2008	442 (66.3)	41.2 ± 5.7	463 (62.9)	Hospital	5
Ekpanyaskul 2010 [64]	Khon Kaen, Thailand	2002–2004	516 (42.0)	46.9 ± 10.6	516 (34.9)	Hospital	6
Ma 2010 [65]	USA	1994–1998	1197 (78.8)	36–64	2015 (79.6)	Community	8
Hahjisavvas 2010 [66]	Cyprus	1999–2005	1103 (25.4)	50–59	1173 (25.1)	Hospital	7
Ozmen 2009 [67]	Istanbul, Turkey	2000–2006	1492 (18.4)	18–70	2167 (27.8)	Hospital	7

**Table 2:** Analysis of the potential modifying effects of other risk factors on the relationship of OC use and BrCa risk.

Variables	Studies, N (Ref.)	OR (95% CI)	*p*	*I*^2^ (%)
Taking OC				
ever vs. never	42 [26,27,28,29,30,31,32,33,34,35,36,37,38,39,40,41,42,43,44,45,46,47,48,49,50,51,52,53,54,55,56,57,58,59,60,61,62,63,64,65,66,67]	1.15 (1.01, 1.31)	0.0358	92.32
The period of OC using				
≤5 years	13 [28,33,37,38,41,48,49,50,51,53,59,62,66]	0.92 (0.77, 1.10)	0.3674	85.86
>5 years		1.05 (0.88, 1.25)	0.5787	82.95
Age of menarche				
≤12 years vs. >12 years	29 [26,27,30,32,33,34,35,36,37,38,39,41,43,44,47,48,49,50,51,52,54,55,56,57,58,61,62,64,66]	1.18 (1.07, 1.31)	0.0016	80.71
Parity				
Nulliparous vs. Parous	30 [26,27,30,34,35,36,37,38,39,41,42,43,45,47,48,49,50,51,52,54,55,56,57,58,61,62,63,65,66,67]	1.22 (1.04, 1.43)	0.0146	89.88
Age at first pregnancy				
≥25 years vs. <25 years	14 [27,35,36,37,39,41,43,49,51,55,62,63,66,67]	1.09 (0.94, 1.25)	0.2599	86.64
≥30 years vs. <30 years	6 [26,48,52,58,64,65]	3.08 (1.10, 8.60)	0.0322	97.49
Breastfeeding				
No vs. Yes	27 [26,29,30,32,33,36,37,38,39,41,43,44,45,46,47,48,50,51,52,54,58,61,62,64,65,66,67]	1.36 (1.13, 1.63)	0.0010	91.81
Menopausal status				
Post- vs. Pre-	22 [26,32,34,35,36,37,39,42,43,44,45,46,47,48,50,52,54,55,58,62,63,64]	1.36 (1.14, 1.63)	0.0007	92.86
Family history of BrCa				
Yes vs. No	29 [27,28,29,30,32,33,34,35,37,39,41,43,44,45,46,48,49,50,51,52,54,55,57,58,61,63,64,66,67]	1.72 (1.32, 2.24)	0.0001	93.07
Body mass index (kg/m^2^)				
≥30 (obesitas) vs. <30	16 [26,27,28,29,32,34,39,41,44,49,51,54,55,57,63,66]	1.19 (0.95, 1.5)	0.1289	90.46
Tobacco smoking				
Yes vs. No	14 [26,28,29,30,36,42,49,52,54,58,62,64,66,67]	1.52 (1.26, 1.83)	0.0000	72.32
Diabetes				
Yes vs. No	4 [26,29,32,39]	1.99 (0.60, 6.62)	0.2605	93.49
